# Neural responses to intention and benefit appraisal are critical in distinguishing gratitude and joy

**DOI:** 10.1038/s41598-020-64720-y

**Published:** 2020-05-12

**Authors:** Guanmin Liu, Zaixu Cui, Hongbo Yu, Pia Rotshtein, Fangyun Zhao, Haixu Wang, Kaiping Peng, Jie Sui

**Affiliations:** 1 Department of Psychology, Tsinghua University, Beijing, 100084 China; 2 Center for Healthy Minds, University of Wisconsin-Madison, Madison, WI 53703 USA; 3 Department of Psychiatry, Perelman School of Medicine, University of Pennsylvania, Philadelphia, PA 19104 USA; 4 Department of Psychology, Yale University, New Haven, CT 06520 USA; 5 School of Psychology, University of Birmingham, Birmingham, B15 2TT UK; 6 Department of Psychology, University of Wisconsin-Madison, Madison, WI 53706 USA; 7 School of Psychology, University of Aberdeen, Aberdeen, AB24 3FX UK

**Keywords:** Prefrontal cortex, Agency

## Abstract

Gratitude and joy are critical for promoting well-being. However, the differences between the two emotions and corresponding neural correlates are not understood. Here we addressed these issues by eliciting the two emotions using the same stimuli in an fMRI task. In this help reception task, participants imagined them in a situation where they need financial aid. Critically, we manipulated the benefactor’s intention to provide help and the value of the benefit. Behaviorally, gratitude was stronger than joy when the benefactor-intention was strong and the benefit-value was low compared to other conditions. In parallel, gratitude activated mentalizing-related (e.g. precuneus) and reward-related regions (e.g. putamen) more strongly than joy in corresponding conditions compared to others. Moreover, gratitude was more negatively (or less positively) encoded in the region associated with mentalizing (i.e. the left superior temporal gyrus) than joy. Multivariate pattern analysis further demonstrated that the modulation patterns of benefactor-intention and benefit-value in mentalizing-related (e.g. precuneus, temporo-parietal junction) and reward-related regions (e.g. putamen, perigenual anterior cingulate/ventromedial prefrontal cortex) could distinguish the two emotions. The findings suggest that benefactor-intention and benefit-value appraisal and their neural correlates are critical in distinguishing gratitude and joy. Direct implications for gratitude interventions were discussed.

## Introduction

Imagine that you are in urgent need of a large sum of money. Your friend John tried diligently to collect money for you with demonstrable concern for your need, but unfortunately failed to raise significant funds. You may not feel joyful about the outcome, but you may still feel grateful to him for his good intentions and pronounced efforts in his assisting you. Gratitude and joy are both positive emotions important to subjective well-being (SWB)^1,2^. They enhance each other at the trait level across time, which is unique among positive affects and may be critical to SWB^2^. Although gratitude and joy are judged by lay people as highly similar^3^, they appear to have different cognitive antecedents and behavioral tendencies according to a prior qualitative study^4^. The differences between the two emotions and corresponding neural correlates, however, remain unclear.

According to the appraisal theories^5–7^, emotions can be differentiated by cognitive appraisals that induce them. Ortony, *et al*.^6^ postulated that joy arose from the appraisal of desirability, i.e. whether the outcome is desirable, which mainly consists of the appraisal of benefit-value; while gratitude from that of both desirability and praiseworthiness, i.e. whether the benefactor is worth praising, which mainly consists of the appraisal of the benefactor’s intention to provide help (referred hereafter as benefactor-intention). According to Ortony, *et al*.^6^, perceived benefit is crucial in inducing joy but not gratitude; on the contrary, perceived praiseworthiness (generous benefactor-intention) is crucial in inducing gratitude, and gratitude can be elicited even when there is no perceived benefit. Taking the scenario above for example, when the benefit-value is low, people still feel grateful because the perceived generous benefactor-intention yields an alternative source of desirability (rewards), which compensates the lack of benefit-value for gratitude. In other words, when the benefit-value is low, generous benefactor-intention yields both sources of praiseworthiness and desirability, which in turn produces gratitude; but this is not the case for joy because its induction relies on the benefit rather than the benefactor-intention. These postulations suggested that benefactor-intention and benefit-value might be two critical factors in distinguishing gratitude and joy.

Abundant research has found that joy or happiness is associated with the reward system (e.g. the ventromedial prefrontal cortex [VMPFC], striatum)^8–10^. On the other hand, research on the neural basis of gratitude is still in its infancy. So far, fMRI studies of gratitude have used the paradigm of imaginary scenario^11,12^ or ostensible interpersonal interaction^13–15^ to induce gratitude. Studies using both paradigms consistently found that gratitude activated reward-related (e.g. VMPFC, perigenual anterior cortex [pgACC], striatum) and mentalizing-related regions (e.g. temporo-parietal junction [TPJ], superior temporal gyrus [STG], posterior cingulate cortex [PCC]).Voxel-based morphometry (VBM) studies^16,17^, on the other hand, revealed that gratitude uniquely related to gray matter volume in regions associated with mentalizing and face perception (e.g. TPJ/posterior superior temporal sulcus [pSTS], fusiform gyrus and inferior temporal gyrus [ITG]) after general positive affect (highly correlated with joy^2^) was controlled for.

Based on these previous findings^4,14–16^ and postulations^6^, the present study aimed to test the critical role of benefactor-intention appraisal in differentiating gratitude and joy and its dependence on the level of benefit-value, using an imaginary scenario paradigm. Participants were instructed to imagine that they were in urgent need of money and then shown various scenarios with a level of their friend’s intention to help and an amount of money given by them. Following each scenario, they were asked to rate their level of gratitude and joy. From previous speculation that benefactor-intention appraisal is crucial in producing gratitude but not joy^6^, we predicted that the effect of benefactor-intention on gratitude and joy would be different (specifically, gratitude would be more sensitive to benefactor-intention than joy would). From the speculation that generous benefactor-intention yields desirability (rewards) that compensates the lack of value (when the benefit-value is low vs. high) for gratitude but not joy^6^, we predicted that the effect of benefit-value on gratitude and joy would be different (specifically, gratitude would be less sensitive to benefit-value than joy would). We also explored whether the effect of benefactor-intention would be contingent on the level of benefit-value, based on the speculation that generous intention may be more salient when the benefit-value is low vs. high.

At the neural level, because benefactor-intention serves as a cue to the benefactor’s intention as well as a source of desirability for gratitude but not joy when the benefit-value is low (vs. high), we predicted that the effects of benefactor-intention, benefit-value and the benefactor-intention by benefit-value interaction on gratitude and joy would activate both mentalizing-related regions (induced by intention appraisal), such as TPJ/pSTS, precuneus and medial prefrontal cortex^18^, and reward-related regions (induced by desirability appraisal), such as striatum, VMPFC and ACC^19^. In addition, we would use both univariate and multivariate parametric analyses to directly compare the effects (i.e. parametric modulator slopes) of benefactor-intention and benefit-value on the neural activity during gratitude and joy events, corresponding to the effects mentioned above. We predicted that the modulator slopes of benefactor-intention and benefit-value could dissociate gratitude from joy in the same regions. Based on previous findings from VBM studies that gratitude related to temporal volumes (TPJ/pSTS, fusiform gyrus, ITG) distinctively after general positive affect was controlled for^16,17^, we predicted that gratitude and joy would show different activation or be represented differently (i.e. different modulation slope by emotion rating) in these regions, especially TPJ/pSTS for its role in mentalizing^18^.

## Methods

### Participants

Thirty Chinese college students (15 females; 19–30 years, mean ± SD = 23.40 ± 2.98) participated in the experiment. All participants were right-handed and had normal or corrected-to-normal vision. All participants gave informed consent. The study protocol was approved by the Institutional Review Board of Tsinghua University, and the experiment was carried out in accordance with the approved guidelines.

## Design and Procedure

In this study, we used scenario paradigm to induce gratitude and joy so that we were able to manipulate benefactor-intention with various levels (rather than simply intentional vs. unintentional) and compare the neural representations of benefactor-intention between gratitude and joy. Previous studies comparing emotions with imaginary scenario paradigm employed different stimuli to induce corresponding emotions^8^, so they could not rule out the possibility that the differences in neural correlates may be just out of stimuli differences. To address this issue, we designed a task with the same stimuli to induce both gratitude and joy.

**Figure 1:**
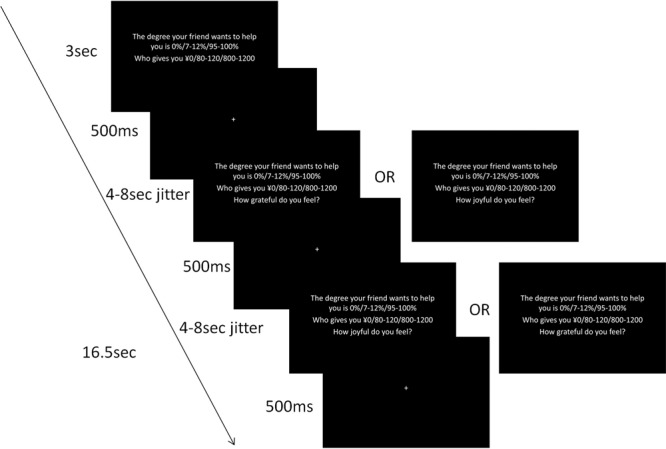
Timeline of experimental procedure. The order of the gratitude rating and the joy rating screen was counterbalanced across runs.

The experiment has a 3 (benefactor-intention: strong, weak, no) ×3 (benefit-value: high, low, zero) ×2 (emotion: gratitude, joy) within-subject design. In each trial, the factors of benefactor-intention and benefit-value were manipulated, while the emotion factor was not manipulated but used only as a cue to which emotion (gratitude or joy) participants were asked to imagine. Specifically, during the experiment, participants were instructed to imagine how they would feel in the situation described on each screen when they were in urgent need of money. As shown in Fig. 1, in each trial, a screen with two lines of text (“The degree your friend wants to help you is X%”, “who gives you ¥Y”) was shown for 3 seconds, followed by a 500 ms fixation. The X% varied from 95% to 100% in the strong benefactor-intention condition (Intention_Strong_), 7% to 12% in the weak benefactor-intention condition (Intention_Weak_), and 0% in the no benefactor-intention condition (Intention_No_). The ¥Y varied from ¥800 to ¥1200 in the high benefit-value condition (Value_High_), from ¥80 to ¥120 in the low benefit-value condition (Value_Low_), and ¥0 in the zero benefit-value condition (Value_Zero_). On the following screen, participants rated how grateful they felt within 4–8 seconds, by pressing the button corresponding to “strongly”, “moderately” or “weakly”. This was followed by a 500 ms fixation. Finally, another screen instructing participants to rate how joyful they feel was shown for 4–8 seconds, also followed by a 500 ms fixation. The order of the two ratings was counterbalanced across runs in one of two orders (A-B-B-A-A-B or B-A-A-B-B-A) evenly distributed across subjects. The duration of the two ratings in each trial was one of the following permutations: 8 s/4 s, 7 s/5 s, 6 s/6 s, 5 s/7 s, 4 s/8 s. As a result, the total duration of each trial was consistently 16.5 s.

The nine conditions (3 benefactor-intention by 3 benefit-value levels) were presented in pseudo-random order to ensure the same level of benefactor-intention or benefit-value was not presented on two consecutive trials. Six runs of twenty trials, including two null trials consisting of asterisks with lengths equaling to those of the stimuli in other trials, were carried out in the task. Note that one out of the thirty participants completed only four runs of the experiment and data from trials where either gratitude or joy rating was missing were excluded from all analyses.

### Neuroimaging data acquisition

Functional MRI was performed on a Philips Achieva 3.0 T TX scanner with a SENSE 8-channel head coil at the Center of Biomedical Engineering, Tsinghua University. T1-weighted structural images were acquired with TR = 8.2 ms, TE = 3.8 ms and flip angle = 8°. The SENSE factor was 2/1.5 for AP/RL, and the acquisition matrix was 256 mm × 256 mm. One hundred and sixty contiguous sagittal slices were acquired with voxel size of 1×1×1mm^3^. T2-weighted functional images were acquired with TR = 2300 ms, TE = 35 ms, and flip angle = 90°. FOV was 240 mm. Voxel size was 2.5 × 2.5 × 3.5 mm^3^. One hundred and forty-five volumes per run were acquired, with 37 slices per volume and slice gap = 1 mm.

### Neuroimaging preprocessing

Imaging data were analyzed using the SPM12 software package (https://www.fil.ion.ucl.ac.uk/spm/software/spm12/). T1 images of each participant were first manually adjusted to match the template for better registration. Motion and time correction were conducted to T2 images with Realignment and Slice-timing, respectively. T2 images were matched to T1 images with Coregistration and segmented into gray matter, white matter and cerebrospinal fluid with New Segmentation. Using DARTEL, gray matter template was built and registered to MNI template. The transformation parameters estimated during unified segmentation were applied to the T2 images with Normalized to MNI (and resampling to 3.0 mm cubic isotropic voxels). T2 images were smoothed with 6.0 mm FWHM Gaussian kernel.

## Univariate activation analysis of neuroimaging data

### Neural correlates of gratitude vs. joy and the interaction effects

To investigate the neural correlates of gratitude vs. joy and the interaction effects, a general linear model (GLM; Model 1) was created for each subject with eighteen predictors for the onsets of all conditions and nuisance regressors for the emotion rating order indicator and the six movement parameters. Out-of-brain voxels were removed by masking with the brain mask of SPM in first-level analyses^20^. Contrast images for each condition were computed for each subject.

To assess the neural correlates of gratitude vs. joy, contrast images of gratitude > joy and joy > gratitude were created for each subject. One-sample t-tests were conducted at the group level. A gray matter mask was created by binarizing SPM’s prior probability gray matter map at the threshold of 0.2 and was applied to all the group-level analyses. All analyses were conducted across the whole brain, with an initial threshold of *p*_uncorr_ < 0.001, and we reported as significant those regions which further passed a cluster-correction for multiple comparisons with *p*_FWE_ < 0.05, using Monte Carlo and 3dClustSim in AFNI 18 (https://afni.nimh.nih.gov/). Clusters passing *p*_FWE_ < 0.05 with an initial threshold of *p*_uncorr_ < 0.005 were reported as trends.

Because we observed the emotion by benefactor-intention, the emotion by benefit-value, and the three-way interaction effects from the behavioral analyses, we defined and created contrast images corresponding to the behavioral results of the interaction effects in each subject as follows:

The emotion by benefactor-intention interaction effect:

Gratitude_(Intention_Strong_ − 1/2Intention_Weak_ − 1/2Intention_No_)

> Joy_(Intention_Strong_ − 1/2Intention_Weak_ − 1/2Intention_No_)

The emotion by benefit-value interaction effect:

Gratitude_(1/2Value_Low_ + 1/2Value_Zero_ − Value_High_)

> Joy_(1/2Value_Low_ + 1/2Value_Zero_ − Value_High_)

The three-way interaction effect:

Gratitude_[(Value_Low/Zero__Intention_Strong_ − Value_High__Intention_Strong_) –

(Value_Low/Zero__Intention_Weak/No_ − Value_High__Intention_Weak/No_)]

> Joy_[(Value_Low/Zero__Intention_Strong_ − Value_High__Intention_Strong_) –

(Value_Low/Zero__Intention_Weak/No_ − Value_High__Intention_Weak/No_)]

where Value_Low/Zero_ = 1/2(Value_Low_ + Value_Zero_), and Intention_Weak/No_ = 1/2(Intention_Weak_ + Intention_No_). The same procedures of one-sample t-tests and voxel-/cluster-wise thresholds mentioned above were applied.

### Comparison of neural representations of emotion rating, benefactor-intention and benefit-value between gratitude and joy

To compare the neural representations of emotion rating, benefactor-intention and benefit-value between gratitude and joy, we constructed another GLM (Model 2) for each subject, which included predictors for the onsets of gratitude and joy rating events, each followed by four parametrically varying modulators in the following order with serial orthogonalization: emotion rating, benefactor-intention in linear function and in quadratic function, and benefit-value. The same nuisance regressors for the emotion rating order indicator and the six movement parameters as in Model 1 were added in the model. Specifically, strong/weak/no benefactor-intention and high/low/zero benefit-value were coded as 1/0/−1, respectively. We added the modulator of benefactor-intention in quadratic function because the results from Model 1 showed that the Intention_strong_ and the Intention_No_ condition equally activated some mentalizing-related regions (e.g. TPJ/pSTS, precuneus; see Table S1) more strongly than the Intention_Weak_ condition, which may be due to the fact that both strong and no benefactor-intention are more salient than a weak one in daily life. Out-of-brain voxels were removed in first-level analyses with the same method as applied in Model 1. Contrast images for each modulator were computed for each subject. Paired-samples t-tests were conducted at the group level to investigate whether neural representations of each modulator differed between gratitude and joy. Average beta values in the significant cluster (the left STG) were extracted for visualization.

### Multivariate pattern analysis of neuroimaging data

To examine whether the neural representations of benefactor-intention and benefit-value can dissociate gratitude from joy by corresponding beta patterns, we applied multivariate pattern analysis to classify the parametric modulator slope patterns in pairs (gratitude vs. joy). We used the unsmoothed beta maps corresponding to modulators of benefactor-intention in linear and in quadratic function and those of benefit-value (in linear function) generated for each participant from Model 2. For each pair of modulators, there are 30 × 2 beta maps in total. Therefore, for either gratitude or joy group, each subject has one beta map associated with corresponding modulator. The values of the voxels within the same gray matter mask mentioned above were extracted to generate a feature vector for each subject. Each voxel value in the beta map was a feature for classifying gratitude and joy groups.

Linear SVM was used to classify gratitude and joy groups based on the selected features. SVMs^21^ are currently the most widely used supervised learning method. The parameter C was set at the default value (C = 1). The LIBSVM toolbox for Matlab was used to perform the linear SVM classification (http://www.csie.ntu.edu.tw/~cjlin/libsvm/)^22^. Leave-one-participant-out cross-validation was used to evaluate the performance of the classifier. In each fold, the two beta images of one subject were left out as the testing sample, and the remaining beta images were used as the training sample. Each feature was linearly scaled to the range of 0–1 across the training dataset, and the scaling parameters were also applied to scale the testing dataset. Within the training sample, a paired two-sample two-tailed t-test was applied to each feature, and the features with significant differences (*p*_uncorr_ < 0.05) were retained^23,24^. It should be noted that this feature selection process was performed on the training set only to avoid over-fitting of the classifier. We trained a classifier using training set and the selected features, and tested it by identifying the category of the testing samples. This procedure was repeated 30 times so that the beta images for each participant in the sample were used as the testing sample once. The classification accuracy was calculated, which was defined as the quantity of beta images that were correctly classified. Also, specificity and sensitivity were computed, which are the proportions of gratitude and joy samples correctly classified, respectively. The permutation test was applied to determine whether the classification accuracy was significantly higher than expected by chance^23,25,26^. We applied the above prediction procedure 1,000 times, each time we permuted the labels across the training samples without replacement. The *p* value of the classification accuracy was the portion of the permutations that showed a higher value than the actual accuracy for the real sample.

Then, we localized the pattern information that contributed to the classification. In each fold, the feature selection was based on a slightly different subset of the data. Thus, the selected features differed slightly from fold to fold. The relevant features were restricted to those selected in every validation fold^23,24^. It is well established that the weight vector of a linear SVM classifier represents the contribution of the features to the classification^27,28^. The discriminative weight for each feature was defined as the average of their absolute weight across all folds. Specially, the absolute value of the weight quantifies the contribution of the corresponding feature to the classification. According to prior literature^29^, we reported clusters with at least 5 voxels that contributed to the prediction.

## Results

### Behavioral results

**Figure 2:**
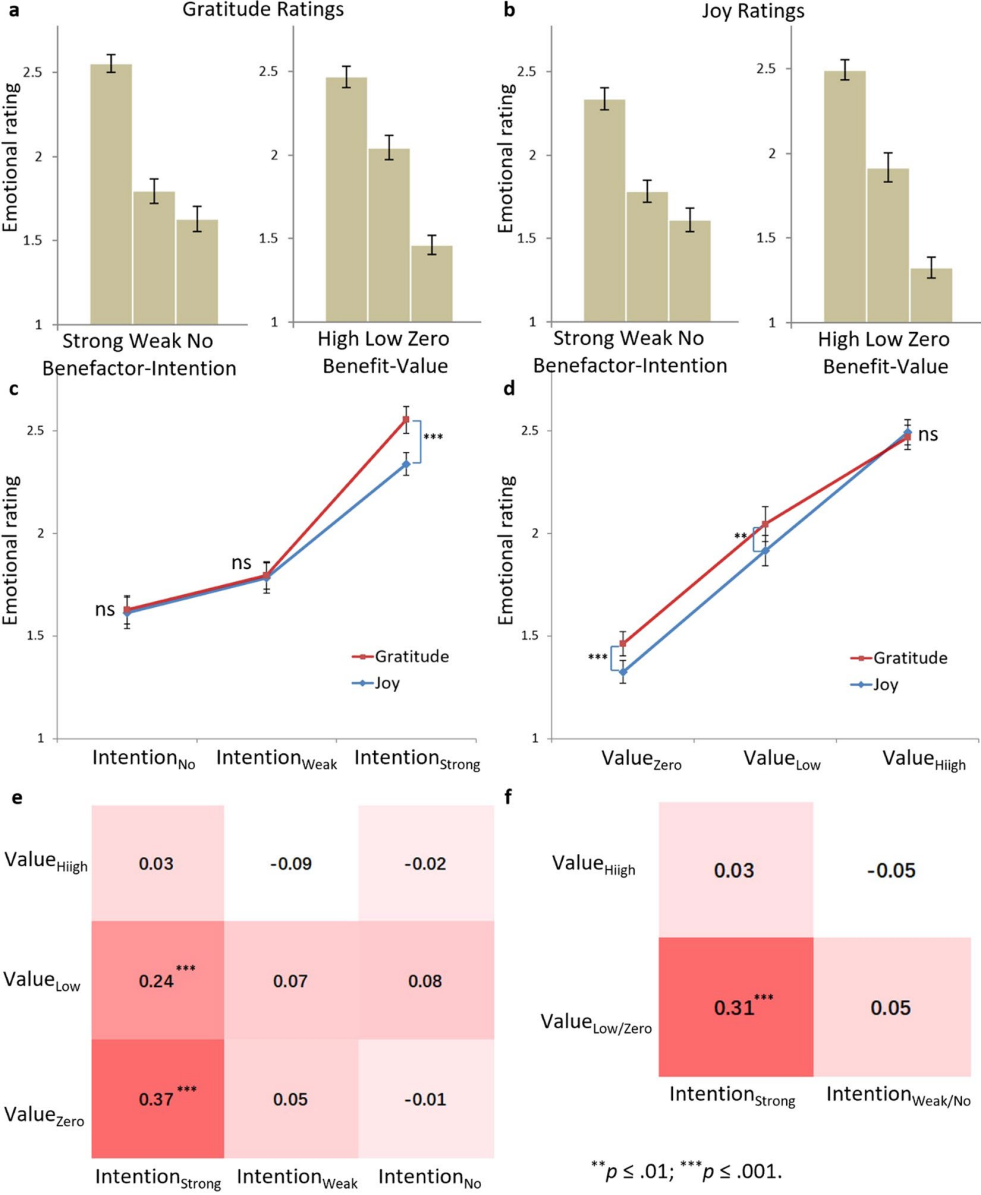
Behavioral results. (**a**) Gratitude ratings for the three levels of benefactor-intention and benefit-value. Error bars represent standard errors in all figures. (**b**) Joy ratings for the three levels of benefactor-intention and benefit-value. (**c**) Emotion ratings in conditions of emotion (gratitude vs. joy) by benefactor-intention. The difference between gratitude and joy rating in the Intention_Strong_ condition was significant. (**d**) Emotion ratings in conditions of emotion (gratitude vs. joy) by benefit-value. The differences between gratitude and joy rating in the Value_Low_ and the Value_Zero_ conditions were significant. (**e**) Mean differences between gratitude and joy rating (positive number means gratitude > joy) in each condition. The deeper the color, the higher gratitude was than joy rating. The differences between gratitude and joy rating in the Value_Low__Intention_Strong_ and Value_Zero__Intention_Strong_ conditions were significant. (**f**) Mean differences between gratitude and joy rating after ratings were averaged across conditions of Value_Low_ and Value_Zero_ as well as those of Intention_Weak_ and Intention_No_. The difference between gratitude and joy rating in the Value_Low/Zero__Intention_Strong_ condition was significant.

To examine whether benefactor-intention and benefit-value have a main effect on gratitude and joy, we conducted benefactor-intention by benefit-value repeated measures ANOVAs with gratitude and joy as dependent variables, respectively. As shown in Fig. 2a,b, the results showed that both had a main effect on gratitude or joy rating (benefactor-intention on gratitude: *F*(2, 58) = 76.31, *p* < 0.001, *η*_*p*_^2^ = 0.73; benefactor-intention on joy: *F*(2, 58) = 68.72, *p* < 0.001, *η*_*p*_^2^ = 0.70; benefit-value on gratitude: *F*(2, 58) = 108.76, *p* < 0.001, *η*_*p*_^2^ = 0.79; benefit-value on joy: *F*(2, 58) = 133.68, *p* < 0.001, *η*_*p*_^2^ = 0.82). Multiple comparisons demonstrated that gratitude and joy ratings for all levels of benefactor-intention or benefit-value were significantly different from each other (gratitude among benefactor-intention levels: *t*(29)s ≥ 3.91, *p*s ≤ 0.001; joy among benefactor-intention levels: *t*(29)s ≥ 4.52, *p*s < 0.001; gratitude among benefit-value levels: *t*(29)s ≥ 7.93, *p*s < 0.001; joy among benefit-value levels: *t*(29)s ≥ 6.96, *p*s < 0.001). Benefactor-intention and benefit-value did not show significant interaction effect on either gratitude or joy.

To investigate whether benefactor-intention or benefit-value has different effects on gratitude and joy, we conducted an emotion (gratitude vs. joy) by benefactor-intention by benefit-value repeated measures ANOVA with emotion rating as dependent variable. The results demonstrated that the main effect of emotion was significant, with the rating of gratitude (1.99 ± 0.28) higher than that of joy (1.91 ± 0.31): *F*(1, 29) = 6.11, *p* = 0.020, *η*_*p*_^2^ = 0.17. Both benefactor-intention and benefit-value showed a main effect on emotion rating (benefactor-intention: *F*(2, 58) = 86.45, *p* < 0.001, *η*_*p*_^2^ = 0.75; benefit-value: *F*(2, 58) = 150.61, *p* < 0.001, *η*_*p*_^2^ = 0.84). As hypothesized, emotion and benefactor-intention showed a significant interaction effect: *F*(2, 34) = 7.66, *p* = 0.003, *η*_*p*_^2^ = 0.21; emotion and benefit-value also showed a significant interaction effect: *F*(2, 58) = 4.42, *p* = 0.024, *η*_*p*_^2^ = 0.13. The interaction effect between benefactor-intention and benefit-value was not significant, but the three-way interaction effect was: *F*(4, 116) = 3.35, *p* = 0.043, *η*_*p*_^2^ = 0.10.

Multiple comparisons showed the difference between gratitude and joy rating in the Intention_Strong_ condition (gratitude: 2.56 ± 0.30; joy: 2.34 ± 0.36) was significant: *t*(29) = 4.38, *p* < 0.001; while those in the Intention_Weak_ and the Intention_No_ conditions were not (Fig. 2c). Likewise, the difference between gratitude and joy rating was significant in the Value_Low_ condition (gratitude: 2.05 ± 0.41; joy: 1.92 ± 0.47): *t*(29) = 2.76, *p* = 0.010; and in the Value_Zero_ condition (gratitude: 1.46 ± 0.31; joy: 1.32 ± 0.33): *t*(29) = 3.98, *p* < 0.001; but not in the Value_High_ condition (Fig. 2d). For the three-way interaction, multiple comparisons showed that gratitude was significantly larger than joy rating in the Value_Low__Intention_Strong_ condition (gratitude: 2.64 ± 0.36; joy: 2.40 ± 0.54): *t*(29) = 3.76, *p* = 0.001; and in the Value_Zero__Intention_Strong_ condition (gratitude: 2.06 ± 0.65; joy: 1.69 ± 0.62): *t*(29) = 3.93, *p* < 0.001; while no significant difference was found in any other condition (Fig. 2e). Based on the results mentioned above, we combine results by averaging ratings across conditions of Value_Low_ and Value_Zero_ as well as those of Intention_Weak_ and Intention_No_, and gratitude was significantly larger than joy rating only in the Value_Low/Zero__Intention_Strong_ condition (gratitude: 2.35 ± 0.44; joy: 2.04 ± 0.54): *t*(29) = 4.19, *p* < 0.001 (Fig. 2f).

## Neuroimaging results of univariate activation analysis

### Neural correlates of gratitude vs. joy and the interaction effects

**Figure 3:**
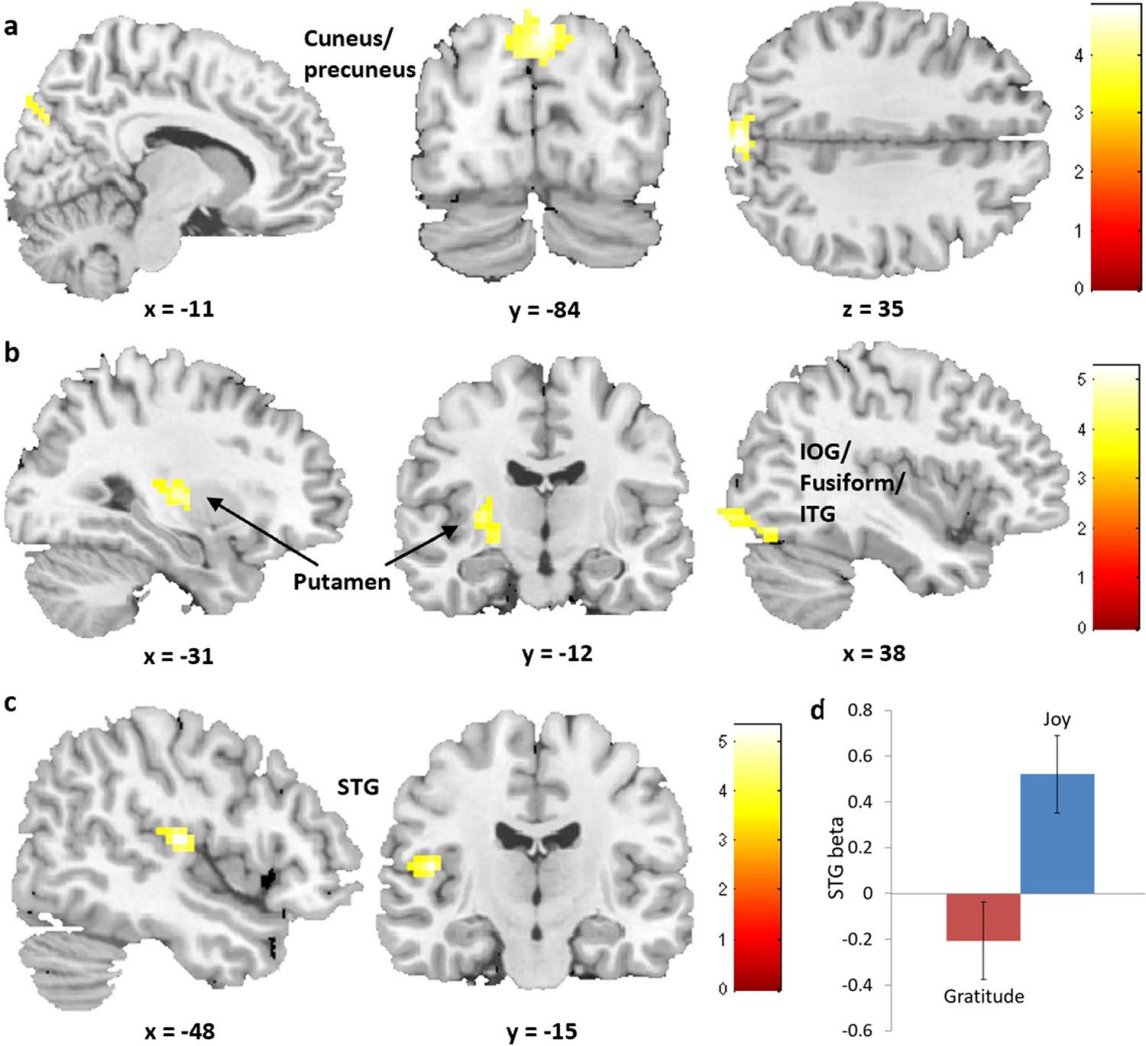
Neuroimaging results of univariate activation analysis. (**a**) Regions activated by the emotion by benefit-value interaction contrast of Gratitude_(1/2Value_Low_ + 1/2Value_Zero_ − Value_High_)> Joy_(1/2Value_Low_ + 1/2Value_Zero_ − Value_High_); (**b**) Regions activated by the three-way interaction contrast of Gratitude_[(Value_Low/Zero__Intention_Strong_ − Value_High__Intention_Strong_) − (Value_Low/Zero__Intention_Weak/No_ − Value_High__Intention_Weak/No_)]> Joy_[(Value_Low/Zero__Intention_Strong_ − Value_High__Intention_Strong_) − (Value_Low/Zero__Intention_Weak/No_ − Value_High__Intention_Weak/No_)]; (**c**) Gratitude and joy were encoded differently in the left STG; (**d**) The average beta values by which neural activity in the left STG was modulated by emotion rating in the gratitude and the joy event.

The neuroimaging analysis failed to show suprathreshold differences in average activation between gratitude and joy. No suprathreshold differences were observed for the emotion by benefactor-intention interaction contrast either. On the other hand, corresponding to the emotion by benefit-value interaction effect in the behavioral results, the contrast showed a significant activation in the left cuneus extending to precuneus, and trends in the precuneus extending to superior parietal lobule, the calcarine extending to precuneus and the left STG (Table 1 & Fig. 3a). Corresponding to the three-way interaction effect in the behavioral results, the contrast showed a significant activation in the left putamen extending to pallidum, the right inferior occipital gyrus (IOG) extending to fusiform/ITG, and a trend in the left dorsolateral prefrontal cortex (Table 1 & Fig. 3b). Results of contrasts between levels of benefactor-intention or benefit-value are shown in Table S1.

**Table 1:** Neuroimaging results of univariate activation analysis (*p*_*uncorr*_ < 0.001 at voxel level, cluster-level *p*_*FWE*_ < 0.05).

Contrast	Region	Cluster Size	Hemisphere	MNI coordinates			*Z*
				x	y	z	
Gratitude vs. Joy	No suprathreshold clusters						
Emotion by intention interaction^a^	No suprathreshold clusters						
Emotion by value interaction^b^	Cuneus/precuneus, BA19/7	81	Left	−6	−87	33	4.10
	Precuneus/SPL, BA6/7/4/5	420^**†**^	Left/Right	0	−48	69	3.91
	Calcarine/precuneus, BA30/29/19	127^**†**^	Left	−6	−45	12	3.77
	STG/MTG, BA41/42/22/40	287^**†**^	Left	−69	−27	3	3.52
Three-way interaction^c^	Putamen/pallidum, BA13	61	Left	−24	−9	−6	4.36
	IOG/fusiform/ITG, BA18/19/37	102	Right	33	−78	−15	4.12
	DLPFC (MFG/IFG), BA46/9/10	270^**†**^	Right	45	36	15	3.69
Gratitude < Joy by linear modulation of emotion rating	STG, BA41/13	96	Left	−48	−15	12	4.38
Gratitude vs. Joy by linear modulation of intention	No suprathreshold clusters						
Gratitude vs. Joy by quadratic modulation of intention	No suprathreshold clusters						
Gratitude vs. Joy by linear modulation of value	No suprathreshold clusters						

^**†**^*p*_*uncorr*_ < 0.005; SPL, superior parietal lobule; STG, superior temporal gyrus; MTG, middle temporal gyrus; IOG, inferior occipital gyrus; ITG, inferior temporal gyrus; DLPFC, dorsolateral prefrontal cortex; MFG, middle frontal gyrus; IFG, inferior frontal gyrus.

^**a**^Gratitude_(Intention_Strong_ − 1/2Intention_Weak_ – 1/2Intention_No_) > Joy_(Intention_Strong_ − 1/2Intention_Weak_ – 1/2Intention_No_).

^**b**^Gratitude_(1/2Value_Low_ + 1/2Value_Zero_ − Value_High_) > Joy_(1/2Value_Low_ + 1/2Value_Zero_ − Value_High_).

^**c**^Gratitude_[(Value_Low/Zero__Intention_Strong_ − Value_High__Intention_Strong_) − (Value_Low/Zero__Intention_Weak/No_ − Value_High__Intention_Weak/No_)] > Joy_[(Value_Low/Zero__Intention_Strong_ − Value_High__Intention_Strong_) − (Value_Low/Zero__Intention_Weak/No_ − Value_High__Intention_Weak/No_)], where Value_Low/Zero_ = 1/2(Value_Low_ + Value_Zero_), and Intention_Weak/No_ = 1/2(Intention_Weak_ + Intention_No_).

### Comparison of neural representations of emotion rating, benefactor-intention and benefit-value between gratitude and joy

Results demonstrated that neural activity in the left STG was more negatively (or less positively) modulated by emotion rating in gratitude than in joy event (Table 1, Fig. 3c,d). The univariate parametric analysis for other modulators did not show suprathreshold differences between gratitude and joy event. Results of one-sample t-tests of each modulator are shown in Table S2.

## Neuroimaging results of multivariate pattern analysis

**Figure 4:**
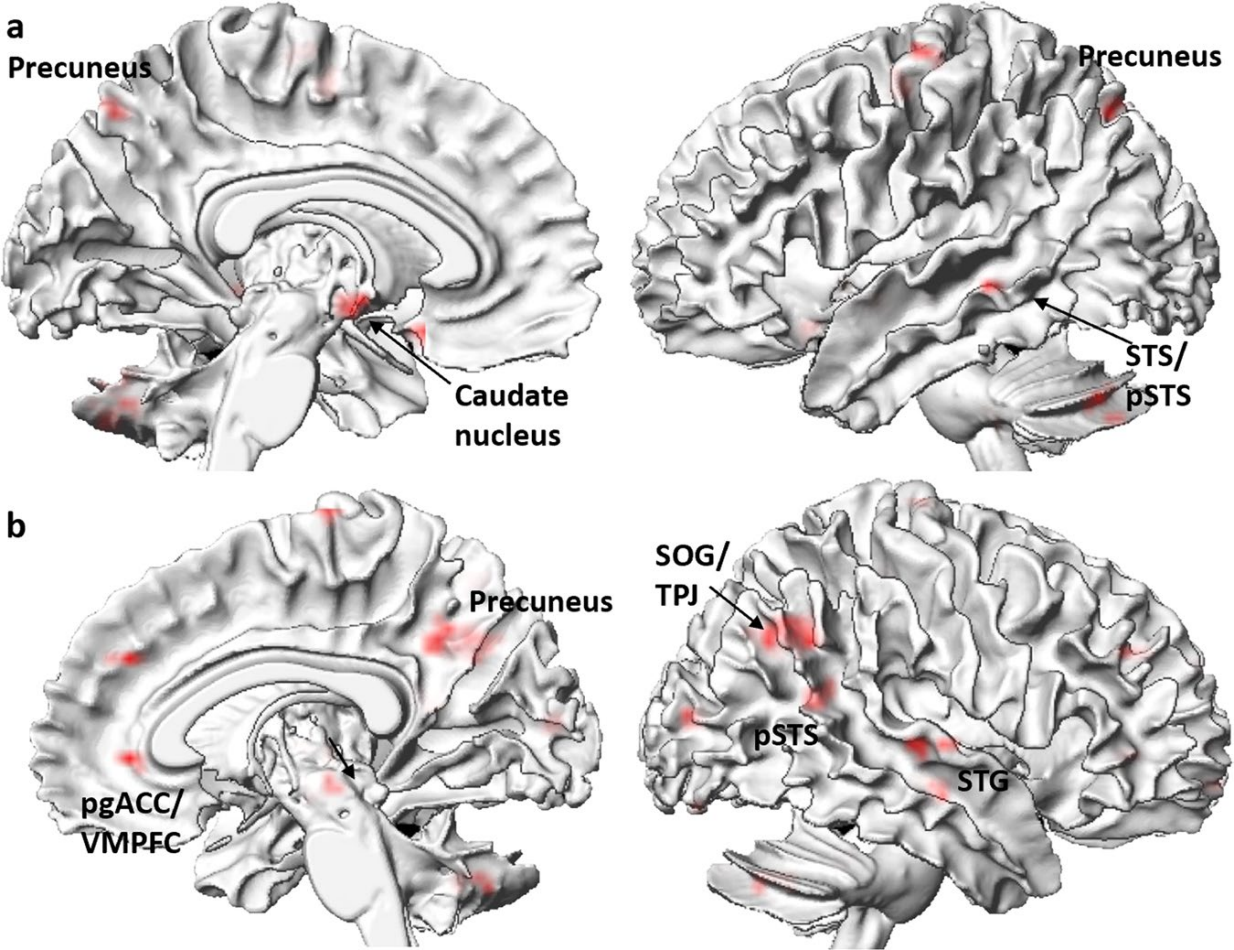
Neuroimaging results of multivariate pattern analysis. (**a**) Modulation patterns of benefactor-intention in mentalizing-related (e.g. precuneus, STS/pSTS) and reward-related regions (e.g. putamen, caudate nucleus) contributed to the discrimination between gratitude and joy; (**b**) Modulation patterns of benefit-value in mentalizing-related (e.g. precuneus, TPJ, pSTS) and reward-related regions (e.g. pgACC/VMPFC, putamen) contributed to the discrimination between gratitude and joy.

According to the leave-one-participant-out cross-validation, the classification accuracy for gratitude vs. joy by linear modulation pattern of benefactor-intention was 68.33% (permutation test *p*_*perm*_ < 0.001; specificity: 66.67%; sensitivity: 70%), that by quadratic modulation pattern of benefactor-intention was 41.67% (*p*_*perm*_ = 0.899; specificity: 33.33%; sensitivity: 50%) and that by linear modulation of benefit-value was 60% (*p*_*perm*_ = 0.024; specificity: 73.33%; sensitivity: 46.67%). The results showed that the classification accuracies for gratitude vs. joy by linear modulation of benefactor-intention and benefit-value, but not quadratic modulation of benefactor-intention, were significantly higher than expected by chance. The weight map representing contributions of all features to the discrimination between gratitude and joy by linear modulation pattern of benefactor-intention included mentalizing-related (e.g. precuneus, superior temporal sulcus [STS]/pSTS) and reward-related regions (e.g. putamen, caudate nucleus) (Table 2 & Fig. 4a). The weight map to the discrimination between gratitude and joy by linear modulation pattern of benefit-value also included mentalizing-related (e.g. precuneus, TPJ, pSTS) and reward-related regions (e.g. pgACC/VMPFC, putamen) (Table 2 & Fig. 4b).

**Table 2:** Neuroimaging results of multivariate pattern analysis (cluster size ≥ 5 voxels).

Region	Cluster Size	Hemisphere	MNI coordinates			*w*
			x	y	z	
**Gratitude vs. Joy by linear modulation pattern of intention**						
Caudate nucleus	13	Left	−3	−3	−9	0.100
Olfactory cortex	5	Left	−3	18	−15	0.098
Cerebellum	12	Left	−30	−72	−42	0.072
Precentral gyrus	5	Left	−27	−12	54	0.069
Putamen/pallidum	8	Right	21	0	6	0.067
STS/pSTS (MTG)	5	Left	−63	−33	−3	0.065
Thalamus	5	Right	12	−15	0	0.058
Cerebellum	5	Right	18	−39	−42	0.057
Cerebellum	8	Left	−27	−69	−27	0.052
Putamen	5	Right	21	3	−6	0.048
Precentral gyrus	7	Left	−33	−15	66	0.047
Caudate nucleus	8	Right	9	9	21	0.042
Hippocampus/amygdala	5	Right	33	−3	−27	0.034
IOG	8	Right	36	−93	−9	0.033
Precuneus/SPL	5	Left	−12	−72	48	0.032
IFG/precentral gyrus	8	Right	42	12	30	0.015
**Gratitude vs. Joy by linear modulation pattern of value**						
MOG	14	Left	−48	−84	6	0.106
pgACC/VMPFC	6	Right	15	39	0	0.096
Cerebellum	5	Left	−27	−45	−33	0.090
Precuneus/cuneus	8	Right	15	−54	42	0.071
SOG/MOG	5	Right	27	−90	12	0.067
Cerebellum	6	Left	−30	−84	−21	0.066
IPL/postcentral gyrus	15	Left	−36	−36	36	0.066
MCC/DMPFC	7	Right	12	45	30	0.064
PCL	5	Left	−3	−27	51	0.064
Cerebellum	13	Left	−39	−81	−27	0.058
Cerebellum	5	Right	15	−90	−15	0.056
Cerebellum	8	Right	21	−63	−36	0.055
PCL/SMA	7	Right	3	−21	72	0.053
Midbrain	8	Left	−3	−21	−9	0.051
SFG	5	Left	−30	57	24	0.051
MFG/IFG	5	Left	−51	45	0	0.049
Postcentral gyrus	5	Left	−39	−30	45	0.047
IFG/precentral gyrus	7	Left	−33	9	30	0.046
OFC (SFG/MFG)	6	Left	−15	66	−9	0.041
SOG/TPJ (AG)	50	Right	36	−63	33	0.037
pSTS (MTG)	8	Right	42	−54	18	0.031
Postcentral gyrus	5	Left	−36	−33	60	0.031
STG	7	Right	57	−12	3	0.027
STG	7	Right	51	−21	3	0.022
Insula	5	Right	42	−15	−9	0.016
MFG	6	Left	−33	63	3	0.016
Precentral gyrus	8	Left	−39	−3	27	0.014
TPJ (AG)	5	Right	45	−63	54	0.008

STS, superior temporal sulcus; pSTS, posterior superior temporal sulcus; MTG, middle temporal gyrus; IOG, inferior occipital gyrus; SPL, superior parietal lobule; IFG, inferior frontal gyrus; MOG, middle occipital gyrus; pgACC, perigenual anterior cingulate cortex; VMPFC, ventromedial prefrontal cortex; SOG, superior occipital gyrus; IPL, inferior parietal lobule; MCC, midcingulate cortex; DMPFC, dorsomedial prefrontal cortex; PCL, paracentral lobule; SMA, supplementary motor area; SFG, superior frontal gyrus; MFG, middle frontal gyrus; IFG, inferior frontal gyrus; OFC, orbitofrontal gyrus; TPJ, temporo-parietal junction; AG, angular gyrus; STG, superior temporal gyrus.

## Discussion

In this study, we investigated whether and how the effects of benefactor-intention and benefit-value on gratitude and joy could be dissociated at both behavioral and neural levels. As hypothesized, benefactor-intention and benefit-value showed a dissociable effect on gratitude and joy. Specifically, gratitude was stronger than joy when the benefactor-intention was strong and the benefit-value was low/zero. From the fMRI results, low/zero compared to high benefit-value activated the left cuneus extending to precuneus more strongly in gratitude than joy, and it additionally activated the left putamen and the right IOG extending to fusiform/ITG more strongly in gratitude than joy when the benefactor-intention was strong compared to weak/no. On the other hand, gratitude was more negatively (or less positively) encoded in the left STG than joy. Multivariate pattern analysis showed that the beta patterns of benefactor-intention and benefit-value in mentalizing-related and reward-related regions were dissociable between gratitude and joy. Our study provided evidence for the critical role of neural responses to benefactor-intention and benefit-value appraisal in differentiating gratitude from joy.

As hypothesized, we found that benefactor-intention showed a dissociable effect on gratitude and joy (strong benefactor-intention induced more intense gratitude than joy), which was consistent with the notion of gratitude as an other-praising or self-transcendent emotion^4,30^ and empirical findings that benefactor-intention appraisal is critical to gratitude^4,14,31,32^. At the neural level, we showed that the modulation patterns of benefactor-intention in mentalizing-related (e.g. precuneus, STS/pSTS) and reward-related regions (e.g. putamen, caudate nucleus) could dissociate gratitude from joy. Previous studies have found gratitude associated with activation or gray matter volume in precuneus/PCC and pSTS/TPJ^14,16^. These regions have been broadly reported to be associated with mentalizing^18,33^. On the other hand, the putamen is a subregion of the striatum, which has been found involved in gratitude in previous studies^11,15^ and widely reported to be associated with reward processing, specifically reward prediction error^19,34^. Taken together, our findings suggest that not only mentalizing but also reward processing evoked by benefactor-intention is critical in differentiating gratitude from joy, consistent with Ortony, *et al*.^6^’s postulations that benefactor-intention serves as a cue to both other’s intention and a source of desirability for gratitude but not joy. According to the theories of gratitude as adaptation for reciprocal altruism and relationship binding^35,36^, the desirability accompanying generous benefactor-intention when gratitude arises may serve as a signal of the value of a high-quality relationship. In line with this, evidence showed that gratitude increased positive relationship ratings with the benefactor, even controlling for other positive emotions^37^.

Intriguingly, the univariate analysis failed to find suprathreshold activations associated with the emotion (gratitude/joy) by benefactor-intention interaction, but the multivariate analysis showed that the parametric modulator slopes of benefactor-intention predicted gratitude and joy beyond chance-level. These results indicated that the dissociation between gratitude and joy by benefactor-intention might lie in modulation slope patterns rather than average activations in regions associated with mentalizing and reward processing. Alternatively, the interaction between emotion and benefactor-intention might be undermined by the significant three-way interaction between emotion, benefactor-intention and benefit-value. Specifically, the results showed that when the benefit-value was low/zero, strong benefactor-intention compensated the lack of benefit-value for gratitude and induced stronger gratitude than joy. These findings suggest that benefactor-intention yields desirability especially when the benefit-value is low, which is consistent with previous postulations^6^. At the neural level, fMRI results showed that the three-way interaction activated the left putamen and the right IOG extending to fusiform/ITG. As mentioned above, the putamen is associated with reward processing^19,34^, and has been found involved in gratitude in previous studies^11,15^. The fusiform gyrus may be associated with social processing. Previous VBM study has shown that gratitude related positively to gray matter volume in the fusiform gyrus^16^, which has been found associated with face perception^38^ and other social cognitions, such as decoding communicative intentions^39^.

We also found an interaction effect between emotion and benefit-value, suggesting that benefit-value also showed a dissociable effect on gratitude and joy. As Ortony, *et al*.^6^ postulated, though both gratitude and joy are influenced by benefit-value, benefactor-intention compensates the lack of benefit-value for gratitude but not joy when the benefit-value is low. Consistent with that, we found that the emotion by benefit-value interaction activated the precuneus and another two trending clusters also including precuneus. Prior meta-analysis^18^ found that precuneus was involved in almost all mentalizing tasks, and might be associated with retrieving situations encoded in memory to match them with current context for selection or inference of appropriate actions and goals. In addition to precuneus, the multivariate pattern analysis showed that gratitude and joy could be dissociated by the modulation patterns of benefit-value in the right TPJ and pSTS, which are another core areas of mentalizing and involved in intention/goal inferences^18,33^. On the other hand, gratitude and joy could also be differentiated by the modulation patterns of benefit-value in pgACC/VMPFC and putamen, which are associated with reward processing^19,40^ and consistently found involved in gratitude processing^12–15,41^.

Finally, we found that gratitude was more negatively (or less positively) encoded in the left STG than joy. Previous research^9,12^ found activation of STG was involved in both gratitude and joy. It was thought to be associated with understanding of social schema and mental states of others from complex social signals in eye gaze, mouth movements and body language^42^. Intriguingly, we found that gratitude showed a trend of negative representation in the region. Considering that the same region also showed a trend of activation in the emotion by benefit-value interaction contrast (i.e. gratitude activated the STG stronger than joy when the benefit-value was low/zero compared to high), it is possible that when the benefit-value is low (and hence lower level of gratitude due to the main effect), individuals process complex social signals more strongly to infer mental states of the benefactor in the gratitude than in the joy event. As a result, compared to joy, gratitude rating may elicit more consistent activation of the STG for inference of the benefactor’s mental states irrespective of the intensity of the emotion. On the contrary, joy rating may elicit activation of the STG stronger when the benefactor-intention and thus the joy rating is high vs. low. These findings further implied the critical role of intention appraisal or mentalizing processes in differentiating gratitude from joy.

The findings also have implications for the potential dynamic mechanisms underlying the relationship between gratitude and joy as well as gratitude interventions. From our findings, benefactor-intention and benefit-value influence both gratitude and joy, which may lay the foundation for their mutual influence. On the other hand, the different effects of benefactor-intention and benefit-value on them and their representation difference in the left STG may offer a compensatory mechanism for individuals to feel good even when the benefit-value is low, which may in turn promote long-term SWB^1,2^. Accordingly, gratitude interventions may be most efficient in boosting SWB by focusing on situations where the benefit-value is low, in which the high desirability yielded by generous intention is most saliently signaled.

While the imaginary task demonstrated a spontaneous emotion production in the laboratory setting, its ecological validity should be examined in the future. Also, other emotions, such as feeling hurt, might be evoked during the task, though they may have been evenly distributed across conditions because of the counterbalance and pseudo-randomization. It would also be interesting to include a wider variety of measures to address the differences between gratitude and joy in the future, such as physiological measures (e.g. vagal tone) and facial expression. In addition, we did not investigate whether gratitude and joy showed different effects on subsequent behavior. As a self-transcendent emotion and one adapted for relationship binding^30,36^, gratitude should show stronger effect on affiliation and prosocial behavior than joy. Future studies should further investigate these topics, as well as the neural mechanisms underlying the dynamic relationship between gratitude and joy.

## Supplementary information

Supplementary information.

## Data Availability

The datasets generated during and/or analyzed during the current study are available from the corresponding author on reasonable request.

## References

[1] Wood AM, Froh JJ, Geraghty AW (2010). Gratitude and well-being: A review and theoretical integration. Clinical psychology review.

[2] Watkins PC, Emmons RA, Greaves MR, Bell J (2018). Joy is a distinct positive emotion: Assessment of joy and relationship to gratitude and well-being. The Journal of Positive Psychology.

[3] Schimmack U, Reisenzein R (1997). Cognitive processes involved in similarity judgments of emotions. Journal of Personality and Social Psychology.

[4] Algoe SB, Haidt J (2009). Witnessing excellence in action: The ‘other-praising’ emotions of elevation, gratitude, and admiration. The journal of positive psychology.

[5] Frijda NH (1988). The laws of emotion. American psychologist.

[6] Ortony, A., Clore, G. L. & Collins, A. *The cognitive structure of emotions*. (Cambridge university press, 1988).

[7] Moors A, Ellsworth PC, Scherer KR, Frijda NH (2013). Appraisal theories of emotion: State of the art and future development. Emotion Review.

[8] Gilead M, Katzir M, Eyal T, Liberman N (2016). Neural correlates of processing “self-conscious” vs.“basic” emotions. Neuropsychologia.

[9] Britton JC (2006). Neural correlates of social and nonsocial emotions: An fMRI study. Neuroimage.

[10] Kühn S, Gallinat J (2012). The neural correlates of subjective pleasantness. Neuroimage.

[11] Zahn R (2008). The neural basis of human social values: evidence from functional MRI. Cerebral cortex.

[12] Fox, G. R., Kaplan, J., Damasio, H. & Damasio, A. Neural correlates of gratitude. *Frontiers in psychology* **6** (2015). 10.3389/fpsyg.2015.01491 PMC4588123 26483740

[13] Kini P, Wong J, McInnis S, Gabana N, Brown JW (2016). The effects of gratitude expression on neural activity. NeuroImage.

[14] Yu H, Cai Q, Shen B, Gao X, Zhou X (2017). Neural substrates and social consequences of interpersonal gratitude: Intention matters. Emotion.

[15] Yu, H., Gao, X., Zhou, Y. & Zhou, X. Decomposing gratitude: representation and integration of cognitive antecedents of gratitude in the brain. *Journal of Neuroscience*, 2944-2917 (2018). 10.1523/JNEUROSCI.2944-17.2018 PMC6596125 29735557

[16] Liu G (2018). Praising others differently: neuroanatomical correlates to individual differences in trait gratitude and elevation. Social Cognitive and Affective Neuroscience.

[17] Zahn R, Garrido G, Moll J, Grafman J (2013). Individual differences in posterior cortical volume correlate with proneness to pride and gratitude. Social cognitive and affective neuroscience.

[18] Van Overwalle F, Baetens K (2009). Understanding others’ actions and goals by mirror and mentalizing systems: a meta-analysis. Neuroimage.

[19] Bartra O, McGuire JT, Kable JW (2013). The valuation system: a coordinate-based meta-analysis of BOLD fMRI experiments examining neural correlates of subjective value. Neuroimage.

[20] Liemburg EJ (2015). Neural correlates of planning performance in patients with schizophrenia—relationship with apathy. Schizophrenia research.

[21] Vapnik, V. *The nature of statistical learning theory*. (Springer science & business media, 2013).

[22] Chang C-C, Lin C-J (2011). LIBSVM: a library for support vector machines. ACM transactions on intelligent systems and technology (TIST).

[23] Dosenbach NU (2010). Prediction of individual brain maturity using fMRI. Science.

[24] Dai Z (2012). Discriminative analysis of early Alzheimer’s disease using multi-modal imaging and multi-level characterization with multi-classifier (M3). Neuroimage.

[25] Cui Z, Su M, Li L, Shu H, Gong G (2018). Individualized prediction of reading comprehension ability using gray matter volume. Cerebral Cortex.

[26] Combrisson E, Jerbi K (2015). Exceeding chance level by chance: The caveat of theoretical chance levels in brain signal classification and statistical assessment of decoding accuracy. Journal of neuroscience methods.

[27] Mourao-Miranda J, Bokde AL, Born C, Hampel H, Stetter M (2005). Classifying brain states and determining the discriminating activation patterns: support vector machine on functional MRI data. NeuroImage.

[28] Cui Z, Xia Z, Su M, Shu H, Gong G (2016). Disrupted white matter connectivity underlying developmental dyslexia: A machine learning approach. Human brain mapping.

[29] Cui, Z., Su, M., Li, L., Shu, H. & Gong, G. Individualized prediction of reading comprehension ability using gray matter volume. *Cerebral Cortex*, 1-17 (2017). 10.1093/cercor/bhx061 PMC6669415 28334252

[30] Stellar JE (2017). Self-transcendent emotions and their social functions: Compassion, gratitude, and awe bind us to others through prosociality. Emotion Review.

[31] Wood AM, Maltby J, Stewart N, Linley PA, Joseph S (2008). A social-cognitive model of trait and state levels of gratitude. Emotion.

[32] Tesser A, Gatewood R, Driver M (1968). Some determinants of gratitude. Journal of personality and social psychology.

[33] Gallagher HL, Frith CD (2003). Functional imaging of ‘theory of mind’. Trends in cognitive sciences.

[34] O’Doherty J (2004). Dissociable roles of ventral and dorsal striatum in instrumental conditioning. science.

[35] McCullough ME, Kimeldorf MB, Cohen AD (2008). An adaptation for altruism: The social causes, social effects, and social evolution of gratitude. Current directions in psychological science.

[36] Algoe SB (2012). Find, remind, and bind: The functions of gratitude in everyday relationships. Social and Personality Psychology Compass.

[37] Algoe SB, Haidt J, Gable SL (2008). Beyond reciprocity: gratitude and relationships in everyday life. Emotion.

[38] Kanwisher N, McDermott J, Chun MM (1997). The fusiform face area: a module in human extrastriate cortex specialized for face perception. Journal of neuroscience.

[39] Wang AT, Lee SS, Sigman M, Dapretto M (2006). Developmental changes in the neural basis of interpreting communicative intent. Social cognitive and affective neuroscience.

[40] Ashar YK, Andrews-Hanna JR, Dimidjian S, Wager TD (2017). Empathic Care and Distress: Predictive Brain Markers and Dissociable Brain Systems. Neuron.

[41] Karns CM, Moore WE, Mayr U (2017). The cultivation of pure altruism via gratitude: a functional MRI study of change with gratitude practice. Frontiers in human neuroscience.

[42] Pelphrey KA, Morris JP, Michelich CR, Allison T, McCarthy G (2005). Functional anatomy of biological motion perception in posterior temporal cortex: an fMRI study of eye, mouth and hand movements. Cerebral cortex.

