# Supplementary material for: Neural responses to intention and benefit appraisal are critical in distinguishing gratitude and joy
Source: Sci Rep. 2020 May 12;10:7864. doi: 10.1038/s41598-020-64720-y (PMC7217870; doi:10.1038/s41598-020-64720-y)
Supplement: Supplementary file 1 — Supplementary information. [file 41598_2020_64720_MOESM1_ESM.docx]

**Supplementary information**

**Neural responses to intention and benefit appraisal are critical in distinguishing gratitude and joy**

Guanmin Liu^1, 2^, Zaixu Cui^3^, Hongbo Yu^4^, Pia Rotshtein^5^, Fangyun Zhao^6^, Haixu Wang^1^, Kaiping Peng^1^, and Jie Sui^7, *^

Author Note

^1^ Department of Psychology, Tsinghua University,

Beijing, 100084, China

^2^ Center for Healthy Minds, University of Wisconsin-Madison,

Madison, WI 53703, USA

^3^ Department of Psychiatry, Perelman School of Medicine, University of Pennsylvania

Philadelphia, PA 19104, USA

^4^ Department of Psychology, Yale University,

New Haven, CT 06520, USA

^5^ School of Psychology, University of Birmingham,

Birmingham, B15 2TT, UK

^6^ Department of Psychology, University of Wisconsin-Madison,

Madison, WI 53706, USA

^7^ School of Psychology, University of Aberdeen,

Aberdeen, AB24 3FX, UK

^*^ Correspondence of this paper should be addressed to:

Jie Sui, [jie.sui@abdn.ac.uk](mailto:jie.sui@abdn.ac.uk)

Table S1. Neuroimaging results from contrasts between levels of benefit-value or benefactor-intention (*p_uncorr_* < .001 at voxel level, cluster-level *p_FWE_* < .05)

| Region | Cluster Size | Hemisphere | MNI coordinates | | | *Z* |
| --- | --- | --- | --- | --- | --- | --- |
|  |  |  | x | y | z |  |
| **Intention_Strong_ > Intention_Weak_** |  |  |  |  |  |  |
| TPJ (AG)/pSTS (MTG), BA39/40/19 | 595 | Left | -54 | -63 | 24 | 6.00 |
| MTG/lOFC/TP, BA21/47/38 | 875 | Left | -60 | -30 | -6 | 5.47 |
| Pre-/postcentral gyrus/SFG, BA6/3/4 | 294 | Left | -42 | -27 | 57 | 5.33 |
| Cerebellum/LG/fusiform, BA18/19/30 | 987 | Right | 27 | -75 | -33 | 5.20 |
| Cerebellum/fusiform, BA18/19 | 116 | Left | -24 | -57 | -24 | 4.82 |
| Precuneus/PCC, BA31/7 | 198 | Left/Right | -12 | -51 | 36 | 4.54 |
| MCC/SMA, BA24/6/23 | 232 | Left/Right | -0 | -18 | 36 | 4.51 |
| PCL/precuneus/SPL, BA4/7 | 68 | Left | -3 | -33 | 78 | 4.27 |
| **Intention_Strong_ > Intention_No_** |  |  |  |  |  |  |
| IOG/fusiform, BA18/19 | 93 | Right | 30 | -81 | -12 | 4.22 |
| **Intention_No_ > Intention_Weak_** |  |  |  |  |  |  |
| Precuneus/cuneus/SOG, BA7/31/18 | 779 | Left/Right | -9 | -90 | 12 | 4.24 |
| TPJ (AG)/pSTS (MTG), BA39/40 | 170 | Left | -51 | -66 | 27 | 3.76 |
| **Value_High_ > Value_Zero_** |  |  |  |  |  |  |
| LG/calcarine, BA17/18 | 251 | Right | 15 | -93 | 0 | 4.97 |
| **Value_Zero_ > Value_Low_** |  |  |  |  |  |  |
| SOG/cuneus, BA18/19 | 146 | Left | -15 | -93 | 21 | 4.79 |
| SMG/pSTS (MTG/STG), BA40/22/39 | 175 | Right | 69 | -48 | 15 | 4.19 |
| Fusiform/ITG, BA37/36/19 | 117 | Left | -24 | -45 | -12 | 4.02 |

LG, lingual gyrus; SOG, superior occipital gyrus; SMG, supramarginal gyrus; MTG, middle temporal gyrus; STG, superior temporal gyrus; ITG, inferior temporal gyrus; TPJ, temporo-parietal junction; AG, angular gyrus; pSTS, posterior superior temporal sulcus; lOFC, lateral orbitofrontal gyrus; TP, temporal pole; SFG, superior frontal gyrus; PCC, posterior cingulate cortex; SMA, supplementary motor area; PCL, paracentral lobule; SPL, superior parietal lobule; IOG, inferior occipital gyrus.

Table S2. Neuroimaging results of parametric modulators (*p_uncorr_* < .001 at voxel level, cluster-level *p_FWE_* < .05)

| Region | Cluster Size | Hemi-sphere | MNI coordinates | | | *Z* |
| --- | --- | --- | --- | --- | --- | --- |
|  |  |  | x | y | z |  |
| **Gratitude by linear modulation of emotion rating (+)** |  |  |  |  |  |  |
| Pre-/postcentral gyrus, BA4/3 | 97 | Left | -33 | -24 | 60 | 4.57 |
| IOG/calcarine/LG/fusiform, BA18/17 | 144 | Right | 27 | -93 | -3 | 4.56 |
| **Joy by linear modulation of emotion rating (+)** |  |  |  |  |  |  |
| Post-/precentral gyrus/IPL, BA4/3/40/6 | 244 | Left | -36 | -27 | 54 | 4.73 |
| LG/IOG/calcarine, BA18/17 | 198 | Left/Right | 36 | -87 | -9 | 4.26 |
| **Gratitude by quadratic modulation of intention (+)** |  |  |  |  |  |  |
| Cuneus/precuneus/SOG, BA31/18/7/19 | 390 | Left/Right | -9 | -51 | 30 | 4.59 |
| STS (MTG/STG), BA21/22 | 161 | Left | -66 | -6 | -6 | 4.21 |
| TPJ (AG)/pSTS (MTG)/MOG, BA39 | 363 | Left | -51 | -66 | 21 | 4.12 |
| lOFC/TP, BA38/47 | 63 | Left | -39 | 24 | -18 | 3.95 |
| Cuneus/SOG, BA7/19 | 99 | Right | 21 | -78 | 27 | 3.78 |
| **Joy by quadratic modulation of intention (+)** |  |  |  |  |  |  |
| Precuneus/SMA/PCL/SPL, BA7/5/6 | 200 | Left/Right | 12 | -24 | 54 | 4.14 |
| pSTS (MTG/STG), BA39 | 123 | Right | 51 | -66 | 12 | 3.93 |
| **Gratitude by linear modulation of value (-)** |  |  |  |  |  |  |
| SOG/cuneus/MOG, BA19 | 224 | Left/Right | -21 | -87 | 39 | 4.98 |

IOG, inferior occipital gyrus; LG, lingual gyrus; IPL, inferior parietal lobule; SOG, superior occipital gyrus; STS, superior temporal sulcus; MTG, middle temporal gyrus; STG, superior temporal gyrus; TPJ, temporo-parietal junction; AG, angular gyrus; pSTS, posterior superior temporal sulcus; MOG, middle occipital gyrus; lOFC, lateral orbitofrontal gyrus; TP, temporal pole; SMA, supplementary motor area; PCL, paracentral lobule; SPL, superior parietal lobule.
